# Complement in patients receiving maintenance hemodialysis: functional screening and quantitative analysis

**DOI:** 10.1186/1471-2369-11-34

**Published:** 2010-12-06

**Authors:** Hiroyuki Inoshita, Isao Ohsawa, Gaku Kusaba, Masaya Ishii, Kisara Onda, Satoshi Horikoshi, Hiroyuki Ohi, Yasuhiko Tomino

**Affiliations:** 1 Division of Nephrology, Department of Internal Medicine, Juntendo University Faculty of Medicine, Tokyo, Japan

## Abstract

**Background:**

The complement system is vital for innate immunity and is implicated in the pathogenesis of inflammatory diseases and the mechanism of host defense. Complement deficiencies occasionally cause life-threatening diseases. In hemodialysis (HD) patients, profiles on complement functional activity and deficiency are still obscure. The objectives of the present study were to measure the functional complement activities of the classical pathway (CP), lectin pathway (LP) and alternative pathway (AP) using a novel method and consequently to elucidate the rates of deficiencies among HD patients.

**Methods:**

In the present study, 244 HD patients at one dialysis center and 204 healthy controls were enrolled. Functional complement activities were measured simultaneously using the Wielisa^®^-kit. The combination of the results of these three pathway activities allows us to speculate which candidate complement is deficient; subsequently, the deficient complement was determined.

**Results:**

All three functional complement activities were significantly higher in the HD patients than in the control group (P < 0.01 for all cases). After identifying candidates in both groups with complement deficiencies using the Wielisa^®^-kit, 16 sera (8.8%) with mannose-binding lectin (MBL) deficiency, 1 serum (0.4%) with C4 deficiency, 1 serum (0.4%) with C9 deficiency, and 1 serum (0.4%) with B deficiency were observed in the HD group, and 18 sera (8.8%) with MBL deficiency and 1 serum (0.5%) with B deficiency were observed in the control group. There were no significant differences in the 5-year mortality rate between each complement-deficient group and the complement-sufficient group among the HD patients.

**Conclusion:**

This is the first report that profiles complement deficiencies by simultaneous measurement of functional activities of the three complement pathways in HD patients. Hemodialysis patients frequently suffer from infections or malignancies, but functional complement deficiencies do not confer additional risk of mortality.

## Background

The complement system is part of the innate immune system comprising at least 40 proteins that collaborate in a complex fashion in the removal of microorganisms and apoptotic cells, but also serves as an opsonin, enhancing and directing adaptive immunity [1,2].

Complement deficiencies associated with several clinical diseases involving impairment of the immune system have been recognized. For example, various reports suggest that complement deficiencies are highly associated with an increased risk for infectious disease [3,4]. Previous studies proposed that complement deficiencies may be a risk factor for severe infections in adults who had no evidence of bacterial/fungal infections in childhood and who have been undergoing chemotherapy or transplant recipients [5,6]. Genetic deficiencies of early components of the classical pathway of complement activation are strongly associated with the development of systemic lupus erythematosus [7]. Recently, the antitumorigenic role of mannose-binding lectin (MBL) was proposed since there was a significantly higher frequency of *mbl2 *genotypes associated with MBL deficiency/insufficiency in ovarian cancer patients than in the age-matched control group [8].

Meanwhile, patients who receive hemodialysis (HD) have various abnormalities of the immune system. HD patients have increased susceptibility to infections and have a high incidence of malignancy, which are possibly linked with the immune response [9,10]. These abnormalities of the immune system may be caused by the uremic state or may occur as a direct consequence of HD therapy. Identification of complement deficiencies in HD patients would lead to a better understanding of the clinical complications and the immune system of patients in the uremic state; however, there is very little information about the deficiencies.

There are three activation pathways of complement, i.e., the classical pathway (CP), the alternative pathway (AP), and the lectin pathway (LP). The CP is triggered by binding of C1q to IgM or IgG antibodies which bind to antigen, which in turn is translated into activation of the serine proteases C1r and C1 s. C1r cleaves the complement components C4 and C2 to form the C3 convertase C4b2a. The AP may start from spontaneous activation of C3 involving factor B (B), factor D (D) and properdin (P). In the LP, MBL acts as the carbohydrate recognition molecule and activates complement association with the MBL-associated serine protease 2 (MASP-2), C1r/C1s-like serine proteases, which in turn catalyze C3 cleavage to C3b, which binds to C4b2a to form C4b2a3b complex (C5 convertase). Finally, the terminal complement complex is formed through the terminal complement pathway (C5, C6, C7, C8 and C9) activated by cleavage of C5 and causes bacteriolysis.

Recently, a commercially available kit (Wielisa^®^-kit, Wieslab, Lund, Sweden) was developed to measure the functional activities of the three complement pathways in parallel for screening for complement deficiencies. In brief, the wells are coated with specific activators of the classical, alternative or lectin pathway. The sample serum is diluted with different specific blockers to ensure that only a specific pathway is activated. Subsequently, C5b-9 is detected with the relevant antibody to the neoantigen expressed during the terminal complement complex formation. The amount of complement activation is correlated with the color intensity and is measured in terms of absorbance, and then the combination of the levels of the three activities can indicate the deficient components.

In the present study, we determined the rates of complement deficiencies among 244 HD patients using the novel functional assay that can allow us to measure the three complement activities simultaneously.

## Methods

### Hemodialysis patients and healthy controls

Serum samples were obtained from 244 HD patients during a hemodialysis session at a single dialysis center (Kasukabe Kisen Hospital, Saitama, Japan) in 2001 and 204 normal healthy controls who had no evidence of renal disease. The hematocrit, hemoglobin, blood urea nitrogen, serum creatinine, total protein, calcium and phosphorus were determined in the serum samples from the HD patients. In an effort to reduce the effect of atherosclerosis by aging, group matching was performed for age when comparing complement activities. The characteristics of the HD patients and healthy controls are shown in Additional file 1. We examined prospectively the mortality rate in terms of complement deficiency among the HD patients from 2001 to 2006. This study was approved by the ethics committee of the Kasukabe Kisen Hospital. All participants gave their informed consent to take part in the study. The name of the current protocol is "Clinical and Observational Study in Hemodialysis Patients", and this research was performed in compliance with the Helsinki Declaration.

### Blood samples

Blood samples from the HD patients were obtained from the arterial side of the patient's arteriovenous fistula before anticoagulant injection at the beginning of an HD session. All samples were immediately centrifuged at 3000 rpm at 4°C for 10 min, separated from the cells and stored at -80°C until use.

### Assay of three complement activation pathways

Measurement of the three functional complement activities was performed using a Wielisa^®^-kit (Wieslab, Lund, Sweden). The enzyme-linked immunosorbent assays (ELISAs) were developed according to the recommendation of the manufacturer. The combination of the three assays for complements can be helpful for detection of complement deficiencies as shown in Additional file 2[11]. The negative control is given the value of 0%, the positive control is given the value of 100%, and subsequently, the values of the sera were expressed as a percentage of the positive control. Low functional complement activities of the CP and AP were defined as serum levels below 5% according to the recommendations of the manufacturer. In the LP via MBL, low activity was also defined as below 5% because there was no individual with MBL deficiency among the individuals with the LP via MBL activation of more than 5% in our study. These definitions were also applied to the healthy control group.

### Detection and measurement of complement components

Serum MBL, MASP-2, B, D and P concentrations were determined by ELISA. MBL, MASP-2 and D ELISA kits were purchased from Antibodyshop (Copenhagen, Denmark), Hycult Biotechnology b.v. (Uden, The Netherlands), and R&D Systems (Minneapolis, MN, US), respectively. B and P ELISA kit**s **were obtained from USCN Life Science Inc. (Wuhan, China). Deficiency was defined as less than 100 ng/mL, 170 ng/mL, 17 mg/dL, 30 ng/mL, and 0.2 mg/dL, for MBL, MASP-2, B, D and P, respectively [12-15]. Serum C2, C5, C6, C7, C8 and C9 concentrations were quantified by nephelometry and serum C3 and C4 concentrations were quantified by turbidimetric immunoassay (SRL Inc., Tokyo, Japan); deficiencies of these complement proteins were defined as less than their respective reference ranges according to SRL Inc (reference ranges: C2:1.6-3.5 mg/dL, C3:86-160 mg/dL, C4:17-45 mg/dL, C5:8-15 mg/dL, C6:2.5-4.5 mg/dL, C7:2.4-4.6 mg/dL, C8:5.5-8.9 mg/dL, C9:2.7-7.3 mg/dL).

### Statistical analysis

All statistical analyses were performed using StatView for Windows, version 5.0 (SAS Institute Inc., Cary, NC). The functional activities of the three complement pathways were measured in sera obtained from the HD patients and healthy controls. The functional activities of CP as well as AP formed normal distributions, and the functional activity of LP formed a roughly bimodal distribution among both the HD patients and healthy controls. The data were expressed as mean ± SD. The Mann−Whitney U-test was used to test the significance of the difference in the three complement pathway activities between the HD patients and healthy controls. Chi-square test was used to test the significance of the difference in frequencies between two groups. A *P*-value of < 0.01 was considered statistically significant.

## Results

### Functional activities of three complement pathways

**Figure 1:**
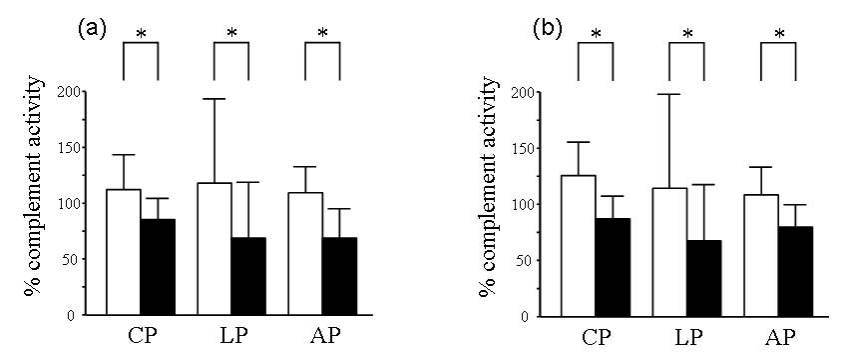
**Comparison of the activities of the classical (CP), lectin (LP) and alternative pathways (AP) in hemodialysis (HD) patients and healthy controls**. White column indicates HD patients, and black column indicates healthy controls. (a) The HD patients enrolled in the present study had significantly higher activity levels of all three complement pathways than the 204 healthy controls. (b) After group matching for age, 64 HD patients also had significantly higher activity levels of all three complement pathways compared with the 56 healthy controls. *P < 0.01; data are shown as the mean ± SD.

The mean CP, LP and AP activities in the HD group were 111.7 ± 30.6%, 117.4 ± 74.9% and 109.3 ± 23.1%, respectively, and those in the control group were 85.6 ± 18.9%, 68.4 ± 50.6% and 68.7 ± 25.6%, respectively. The levels of all three functional complement activities were significantly higher in the HD patients than in the control group (P < 0.01 for all cases; Figure 1a). After group matching for age, the levels of all three functional complement activities were still higher in the HD patients than in the control group (CP, 125.8 ± 33.2% vs. 90.1 ± 19.8%; LP, 115.5 ± 82.0% vs. 67.1 ± 51.1%; AP, 109.9 ± 22.9% vs. 81.0 ± 19.0%; P < 0.01 for all cases; Figure 1b). Moreover, upon comparison of the levels of the three functional complement activities between HD patients who used modified cellulose membrane (n = 35) and HD patients who used polysulfone membrane (n = 29) in the age-matched group, there were no significant differences (data not shown).

### Complement deficiencies

In the HD group, 27 (11.1%) of the 244 samples had low functional activity of one or more complement pathways: 22 (9.0%) had a combination of low LP and normal CP and AP activities, and 3 (1.2%) had a combination of low CP and LP and normal AP activities (Additional file 3). Moreover, 1 (0.4%) had a combination of low functional activity of all three pathways, and 1 (0.4%) had a combination of low AP and normal CP and LP activities. In the control group, 32 (15.7%) of the 204 samples had low functional activity of one or more complement pathways: 31 (15.2%) had a combination of low LP and normal CP and AP activities, and 1 (0.5%) had a combination of low AP and normal CP and LP activities.

To determine which complement was deficient among the HD patients and healthy controls, further experiments were performed according to the combination of the low complement pathway activities (Additional file 2). Additional file 4 Additional file 5 and Additional file 6 show the concentrations of the individual complement proteins in each group. In the HD group, there were 16 patients (6.6%) with MBL deficiency but with sufficient MASP-2 among the 22 patients who presented with low LP and normal CP and AP activities, 1 patient (0.4%) with C4 deficiency but with sufficient C2 among the 3 patients who presented with low CP and LP and normal AP activities, 1 patient (0.4%) with C9 deficiency but with sufficient C5, C6, C7 and C8, who was the only patient presenting low activities of all three pathways, 1 patient (0.4%) with B deficiency but with sufficient D and P, who was the only patient presenting with low AP and normal CP and LP activities. In the control group, 18 individuals (8.8%) with MBL deficiency and 1 individual with B deficiency (0.5%) were observed. There were no significant differences in the rates of all complement deficiencies in the present study between the HD group and control group (Additional file 7).

### Outcomes of the hemodialysis patients

The five-year mortality rate from all causes among the HD patients with MBL, C4 or C9 deficiency from 2001 to 2006 was investigated. During this period, 3 deaths were observed among the 16 patients with MBL deficiency, of which 1 was caused by heart failure, 1 was caused by hepatocellular carcinoma and 1 was caused by intracerebral hemorrhage. No deaths were observed among the individuals with C4, C9 and B deficiency, respectively. Although the five-year mortality rate of each complement-deficient group was lower than that of the complement-sufficient group, there were no significant differences between each complement-deficient group and the complement-sufficient group (Additional file 8).

## Discussion

The complement system is increasingly recognized to have both a protective role and a pathogenic role in the maintenance of renal physiology [16-19]. In this study, we focused on the intricate functions of the immune system including complements especially in patients with end-stage renal disease. Hemolytic assays (e.g., CH50 or AH50) are widely performed to assess complement activity and detect complement deficiency, but there are some problems in terms of cost and effectiveness. A novel ELISA kit, the Wielisa^®^-kit, was recently developed to assess the three complement pathways independently and in parallel. Microtiter plates were coated with each ligand that causes the activation of each complement pathway, and the readout is incorporation of C9 in the terminal complement complex, C5b-9. Subsequently, certain combinations of the three results for functional complement activity can allow us to speculate possible deficient components more easily than the hemolytic assays.

The first important finding in the current study was that the rates of low functional complement activity and complement deficiency did not significantly differ between the HD patients and healthy controls, and the mortality rate did not significantly differ between the complement-deficient group and complement-sufficient group among the HD patients. Complement deficiency is a possible risk factor for infections or malignancy as described above. However, complement deficiencies were not associated with mortality from all causes among the HD patients in our study. These findings might be explained by the beneficial aspect of HD that the patients need to visit a medical facility three times a week and would undergo examinations immediately if they feel sick. Unfortunately, data on hospitalizations in the complement-sufficient group were not available and for this reason, we cannot study the relationship between complement deficiencies and hospitalization for infection. As far as the available data, the total number of admissions for all causes among the HD patients with MBL deficiency were 30, and 7 of them were hospitalized due to infection: 3 had acute enteritis, 1 had acute bronchitis, 1 had pneumonia, 1 had tuberculosis and 1 was unknown. The total number of admissions for all causes in the patient with C4 deficiency was 8, and 2 of them were for infections: 1 had acute bronchitis and 1 had influenza. The patient with C9 deficiency and the patient with B deficiency were not hospitalized during the study period. With regard to the detection of the B deficiency, the Ouchterlony method was also performed before measurement of B concentrations by ELISA. Although B levels in sera of the patient and control with low AP activity were very low based on ELISA, the precipitate line could be seen by observing between the anti-B polyclonal antibody and the samples from the results of the Ouchterlony method. This discrepancy may be explained by the fact that ELISA antibodies recognized different epitopes from those recognized by the Ouchterlony method antibody. In the present study, we weighted the ELISA results heavily and defined both the patient and the control as B deficient because these results, including results from the Wielisa^®^-kit indicated the possibility that the two cases had B structural abnormalities or genetic disorders.

The second finding in the current study was that individuals on HD had significantly higher levels of functional complement activity of all three pathways than healthy controls. In addition, we performed group matching to reduce the effect of aging because recent evidence pointed to an association between excessive complement activity and many diseases relevant to aging such as atherosclerosis or lipid metabolism [20,21], and the activity levels of all three pathways were also significantly higher in the HD patients than in the controls. These results suggest that the concentration of C3 and the late complement components (C5, C6, C7, C8 and C9) may be increased in HD patients with the high complement activities. These possibilities are interesting because the sera of HD patients in present study were obtained before HD, not after HD. Many research groups report that dialysis membranes cause varying degrees of complement activation during HD [22], and the concentrations of C3 and late complement components in sera obtained before HD have been studied in a few reports [23]. Measurement of the concentration of C3 and the late complement components before and after HD should be conducted in a large number of HD patients. One potential explanation for the uncontrolled complement activities is that complement regulatory proteins in the serum may be reduced in the patients. To the best of our knowledge, there are no reports on the association between HD and various serum regulatory proteins. A few reports investigated only erythrocyte complement receptor type 1 (E-CR1) among HD patients as follows: recombinant human erythropoietin improved anemia and increased the level of E-CR1 [24], and a low level of E-CR1 was associated with poor prognosis in HD patients [25]. Meanwhile, deficiency of complement regulatory proteins often occurs along with decreased levels of other complement proteins. For example, C1 esterase inhibitor (C1-INH) deficiency, which is one of the important risk factors of hereditary angioedema (HAE), is associated with an increased cleavage of C4 by C1, which results in low C4 levels during the attack. Three HD patients with low CP and LP and normal AP activities in our study may also have had low C4 level due to C1-INH deficiency, but we could not measure the complement regulatory protein because we did not have enough serum. Examination of these patients including family history did not reveal symptoms that suggested HAE, such as recurrent and transient swelling of face, extremities and airway. Complement regulatory proteins in serum should be studied in the near future to clarify complement-mediated inflammatory manifestations.

## Conclusions

The present study is the first to demonstrate the functional activities of three complement pathways in parallel and the rates of several complement deficiencies among HD patients. Although complement deficiency was suggested to be partially responsible for infection or malignancy, the results of our study suggest that complement deficiencies do not play a role in mortality in HD patients. Nevertheless, these results also suggest that complement deficiencies exist among HD patients as well as healthy individuals, and HD patients with complement deficiency shall be observed more carefully.

## Competing interests

The authors declare that they have no competing interests.

## Authors' contributions

HI carried out complement functional activity, quantitative analysis, all statistical analysis and wrote the manuscript. IO second principal investigator advised on the study and reviewed the manuscript. GK recruited controls for the study. MI carried out ELISA to detect MBL deficiency and recruited controls for the study. KO carried out ELISA to measure properdin concentration. SH participated in the design of the study. HO recruited HD patients for the study, and participated in the design of the study. YT primary principal investigator advised on the study. All authors read and approved the manuscript.

## Pre-publication history

The pre-publication history for this paper can be accessed here:

http://www.biomedcentral.com/1471-2369/11/34/prepub

## Supplementary Material

Additional file 1 **Characteristics of the hemodialysis patients and healthy controls**, Table S1. Click here for file

Additional file 2 **Differential diagnosis panel based on the results of the three complement pathway activities**, Table S2. Click here for file

Additional file 3 **Number of individuals with low complement activity in the hemodialysis and control groups**, Table S3. Click here for file

Additional file 4 **Concentrations of individual complement proteins in hemodialysis patients with low complement activity**, Table S4. Click here for file

Additional file 5 **Concentrations of individual complement proteins in hemodialysis patients with low complement activity**, Table S5. Click here for file

Additional file 6 **Concentrations of individual complement proteins in healthy controls with low complement activity**, Table S6. Click here for file

Additional file 7 **Frequencies of complement deficiencies among the hemodialysis patients and healthy controls**, Table S7. Click here for file

Additional file 8 **Mortality rate among patients with complement deficiency**, Table S8. Click here for file
